# Protective effect of traditional Chinese medicine on non-alcoholic fatty liver disease and liver cancer by targeting ferroptosis

**DOI:** 10.3389/fnut.2022.1033129

**Published:** 2022-10-18

**Authors:** Qiongbo Wu, Zihao Chen, Yi Ding, Yunting Tang, Yawei Cheng

**Affiliations:** ^1^Hainan Provincial Hospital of Traditional Chinese Medicine, Haikou, China; ^2^Hainan Clinical Research Center for Preventive Treatment of Diseases, Haikou, China; ^3^Food Science and Technology Center, National University of Singapore (Suzhou) Research Institute, Suzhou, China

**Keywords:** traditional Chinese medicine, active ingredient, ferroptosis, non-alcoholic fatty liver disease, protective effect

## Abstract

Non-alcoholic fatty liver disease (NAFLD) is a chronic liver disease with high incidence and is closely related to metabolic syndrome. If not controlled, it may eventually become hepatocellular carcinoma (HCC). Ferroptosis, a non-apoptotic form of programmed cell death (PCD), is closely related to NAFLD and HCC, and the mechanisms of action involved are more complex. Some studies have demonstrated that many drugs inhibit ferroptosis and protect liver steatosis or carcinogenesis. The role of Traditional Chinese Medicine (TCM), especially herbs or herbal extracts, has received increasing attention. However, there are relatively few review articles on the regulation of NAFLD by TCM through ferroptosis pathway. Here, we summarize the TCM intervention mechanism and application affecting NAFLD/NAFLD-HCC *via* regulation of ferroptosis. This article focuses on the relationship between ferroptosis and NAFLD or NAFLD-HCC and the protective effect of TCM on both by targeting ferroptosis. It not only summarizes the mechanism of early prevention and treatment of NAFLD, but also provides reference ideas for the development of TCM for the treatment of metabolic diseases and liver diseases.

## Introduction

Liver dysfunction leads to metabolic disorders and ultimately endangers personal health (1). For example, non-alcoholic fatty liver disease (NAFLD) is a manifestation of metabolic syndrome in the liver (2). Simple steatosis occurs when the intrahepatic fat content is > 5% due to of non-alcoholic or other secondary factors, and it can be further developed into non-alcoholic steatohepatitis (NASH). Nearly half of patients with NASH have the probability to develop liver fibrosis, cirrhosis, or even hepatocellular carcinoma (HCC) (3). Approximately 25% of the global population suffer from NAFLD, and the incidence is gradually increasing (4). It is important to note that, in order to reflect the mechanisms of metabolic dysfunction and hepatic steatosis in patients more accurately, NAFLD has been gradually renamed as metabolic associated fatty liver disease (MAFLD) in the recent years (5, 6). While cirrhosis was previously thought to be a major risk factor for the development of HCC, up to 50% of NAFLD related-HCC occurs in patients without cirrhosis as opposed to virus-driven HCC. This group is also often neglected and diagnosed at an older age and at an advanced stage of HCC (7, 8). Hester et al. conducted a cross-sectional study of 13,648 HCC patients and determined that NAFLD was the leading cause of HCC in both the inpatient and outpatient populations, accounting for 32.07 and 20.22% of all cases, respectively (9).

NAFLD is a chronic progressive lesion involving in inflammation, oxidative stress, insulin resistance, and imbalances in lipid metabolism (10, 11). However, the specific underlying mechanisms are not clear, and no definite treatment criteria have been established (12). Cell death determines pathological processes such as liver inflammation, fibrosis, and even transformation (13). Furthermore, hepatocyte ballooning and death can be aggravated by lip toxicity, oxidative stress, organelle dysfunction, or inflammatory response (14). More seriously, NAFLD may gradually transition to NAFLD-HCC status. Therefore, it is necessary to explore strategies to prevent NAFLD to reduce the probability of progression. Programmed cell death (PCD) is a dominant process, which forms part of the core of the complete growth of eukaryotes and plays a regulatory role in NAFLD (15), including apoptosis, necroptosis, autophagy, entosis, paraptosis, pyroptosis, etc. The PCD pathways mentioned above may be activated at different stages of NAFLD, and the key effector molecules involved are also the focus of attention when developing therapeutic agents for NAFLD. Therefore, targeting the modulation of the PCD pathway is an effective approach to prevent or treat NAFLD/NAFLD-HCC (16–18).

Several studies have demonstrated that ferroptosis plays a crucial role in the occurrence of NAFLD (19–21). Inhibition of ferroptosis improves pathophysiology of metabolic-related diseases and is a potential pathway and effective strategy for the prevention and treatment of NAFLD (22, 23). Traditional Chinese medicine (TCM) has the advantages of multi-target, multi-channel, structural stability and high safety (24, 25). And a variety of natural molecules based on TCM such as artemisinin, baicalein, and salvia have been found to be valuable in tumors and nervous system diseases by intervening with ferroptosis (26). However, there is a lack of systematic review of the mechanism of action and clinical application of TCM interventions on ferroptosis affecting the NAFLD disease spectrum. Therefore, we take it as our focus to summarize the association between ferroptosis and NAFLD/NAFLD-HCC, and summarize the intervention mechanisms and applications of TCM that have been reported previously, providing ideas and information for the future development of herbs or herbal extracts for the prevention and treatment of NAFLD/NAFLD-HCC.

## Ferroptosis and non-alcoholic fatty liver disease/non-alcoholic fatty liver disease-hepatocellular carcinoma

### Ferroptosis

**FIGURE 1:**
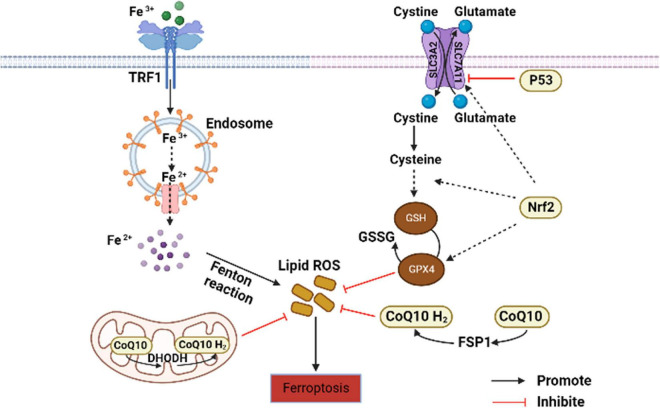
Core regulation of ferroptosis (16). The pathways primarily divided into two based on iron metabolism and oxidative stress. CoQ10, coenzyme Q10; DHODH, dihydroorotate dehydrogenase; FSP1, ferroptosis suppressor protein 1; GSH, glutathione; GPx4, GSH peroxidase 4; GSSG, oxidized glutathione; Nrf2, nuclear factor erythroid 2-related factor 2; ROS, reactive oxygen species; TFR1, transferrin receptor 1.

Ferroptosis is a non-apoptosis with a completely different morphology. Condensation of the cell membrane without affecting membrane integrity, blistering of the plasma membrane, increased density of the mitochondrial membrane, reduction or disappearance of the mitochondrial crest, and rupture of the mitochondrial outer membrane are typical characteristics of ferroptosis (27). It is often accompanied by complex networks of genes, proteins, and metabolisms (Figure 1) (16), which means that it is associated with multiple mechanisms of occurrence. Iron metabolism imbalance, lipid peroxidation and the System Xc-/GSH/GPx4 axis imbalance are the “three hallmarks” (28). Iron is well known to have two different valence states that can undergo redox reactions *in vivo*. The ferroptosis-sensitive cellular transferrin receptor 1 (TFR1) can transports Fe^3+^ into cells for reduction to Fe^2+^, which is stored in the intracellular unstable iron pool in the form of an iron storage protein complex consisting of a ferritin light chain polypeptide, and a ferritin heavy chain polypeptide in the presence of divalent metal ion transport protein 1 (DMT1). However, when excess free Fe^2+^ is present in the cell, ferritin is recruited and solubilized by specific cargo receptor recognition, and the released excess Fe^2+^ in turn increases the formation of hydroxyl radicals through the Fenton reaction, inducing reactive oxygen species (ROS) production and increased susceptibility to cellular ferroptosis (29).

Lipid peroxidation is an important factor driving ferroptosis. Polyunsaturated fatty acids (PUFAs) contain easily extractable diallyl hydrogen atoms, making it sensitive to lipid peroxidation and one of the essential elements for the occurrence of ferroptosis (30). Hydrogen of PUFAs is acquired by hydroxyl groups to form carbon-centered lipid atom groups (L-), and O_2_ reacts rapidly with L- to produce lipid peroxidation atom groups (LOO-). Lipidomic results suggest that phosphatidylethanolamine (PE) containing arachidonic acid (AA) is the key membrane phospholipid for the occurrence of oxidation-driven ferroptosis (31). Acyl coenzyme A synthase long chain family member 4 (ACSL4) and Lys phosphatidylcholine acyltransferase 3 (LPCAT3) are involved in PE biosynthesis, activating PUFA and forming PUFA-PE. The loss of ACSL4 and LPCAT3 depletes substrates for lipid peroxidation and increases inhibition of ferroptosis. Eventually PUFA-PE further promotes ferritic oxidation catalyzed by lipoxygenase (LOX) (32).

System Xc- is a heterodimeric cell surface amino acid reverse transmitter. Extracellular cystine will be transported into cell and reduced to cysteine for the synthesis of glutathione (GSH) (33). GSH is the major antioxidant in mammals and is a cofactor of GPx4. If the level of GSH is compromised by System Xc-, ROS will accumulate and ferroptosis will be initiated by GPx4 with reduced activity (34).

In addition to the above typical pathways that regulate cellular susceptibility to ferroptosis, several others also play a role. P53 is a tumor suppressor gene that inhibits System Xc- uptake by downregulating SLC7A11, affecting GPx4 activity and ultimately inducing ferroptosis (35). Nuclear factor erythroid-2-related factor-2 (Nrf2) is an important antioxidant regulator that promotes the HO-1, GSH, and GPx4 expression in the downstream, eliminates ROS accumulation in the liver, and reduces malondialdehyde (MDA) levels (36). Meanwhile, protein genes responsible for encoding GSH synthesis, such as SLC7A11, GCLC/GLCM, and GSS are all target genes of Nrf2 (37). The latest research has found that dihydroorotate dehydrogenase (DHODH) in mitochondria can regulate ferroptosis through a GSH-independent pathway, which provides a new idea for precise targeted regulation of ferroptosis (38). Therefore, the pathways regulating ferroptosis susceptibility are more abundant and are considered to have important implications in the pathogenesis, treatment or drug development, and specific studies may be more beneficial for its comprehensive understanding.

### Ferroptosis in non-alcoholic fatty liver disease/non-alcoholic fatty liver disease-hepatocellular carcinoma

Metabolic changes and hepatocyte lip toxicity caused by the ectopic accumulation of free fatty acids (FFAs) in the liver are considered to be the principal causes of liver injury in patients with NAFLD (39). However, the mechanisms that drive simple steatosis to NASH, fibrosis and even cirrhosis and HCC are still not fully understood. Whereas the liver is an important organ for iron storage, and the content of iron and lipid ROS in the dysfunctional liver are significantly increased. Serum ferritin is usually showing a high level in patients with NAFLD, which is associated with intrahepatic iron accumulation (40). Insulin resistance is one of the key causative factors of NAFLD, and iron accumulation in the body can also interfere with the function of Islet β Cells, affecting insulin synthesis and secretion, and resulting in insulin resistance (41, 42). Various results suggest that iron metabolism and lipid peroxidation, as the main links in ferroptosis, are closely related to the pathogenesis of NAFLD.

Notably, as researches continue, it has been found that ferroptosis in the different stages of NAFLD may variable. Early results suggested that in addition to iron, levels of the lipid peroxide MDA, 4-NHE, are simultaneously elevated in NAFLD patients (43). Vitamin E, the ferroptosis inhibitor, can reduce lipid peroxidation and improves liver injury (44). Subsequently, Tsurusaki et al. reported that ferroptosis in hepatocytes and intrahepatic macrophages may be the incentive for early simple steatosis and the progression from NAFLD toward to NASH: hepatocyte ferroptosis precedes apoptosis during the initial stages of NAFLD in model mice, leading to liver damage, immune cell infiltration, and inflammatory response (19). Contrastingly, ferroptosis inhibition results in reduced liver injury inflammatory and lipid peroxidation (19). This report provides the first clear insight into the relationship between ferroptosis and NAFLD, and contrasts the differences among hepatocyte ferroptosis, apoptosis and necrosis. Li et al. found that AA metabolism enhanced lipid ROS accumulation, and key regulatory factors of iron metabolism significantly were increased in MCD-induced NASH mice (21). However, these changes occurred after administration of a ferroptosis inhibitor, ferrostatin-1 (21). Increased Fe^2+^ and AA may act jointly to promote lipid peroxidation and NASH. Excessive Fe^2+^ impairs the function of pancreatic β cells and liver cells through oxidative stress and mitochondrial injury, leading to insulin resistance and affecting NAFLD development (41). Other studies have shown that ferroptosis may exacerbate the early inflammatory, oxidative stress, and cell damage in NASH (45). Further progression of NASH can lead to the development of liver fibrosis in patients. The key to the development of liver fibrosis is the activation of hepatic stellate cells (HSC). And HSCs are abundant in iron. Induce activation of HSCs promotes the accumulation of Fe^2+^, elevates ROS levels, and leads to ferroptosis (46). In addition, HSCs contain the ferroptosis regulator P53, ELAV-like protein 1 (ELAV1) and zinc finger monoprotein 36 (ZFP), which have been reported to be effective targets for fibrosis prevention (47, 48). As previously mentioned, P53 inhibits SLC7A11 and reduces GPx4 activity, leading to ferroptosis in HSC. The p62-kelch-like ECH associated protein 1 (Keap1)- Nrf2 antioxidant signaling pathway is more frequently engaged in HCC, which is also involved in the regulation of ferroptosis. Inhibition or knockdown of Nrf2 enhanced erastin- or sorafenib-induced ferroptosis in HCC *in vitro* and *in vivo* (49). Non-coding RNAs (ncRNAs) are responsible for the regulation of tumorigenesis through various biological processes. Among them, microRNA (miRNA) regulates GSH, Fe levels, Nrf2 and ROS to regulate ferroptosis and inhibit cancer development (50, 51). LncRNAs mainly act as the regulatory factors of transcription factors in the nucleus or as miRNAs of sponges in the cytoplasm to regulate ferroptosis (52). In conclusion, ferroptosis is pivotal in the occurrence and progression of the NAFLD disease spectrum. However, unlike the early two stages, the promotion of cellular ferroptosis may be beneficial for liver fibrosis and HCC. This may also be a perspective to distinguish the severity of NAFLD.

## Intervention effects of herbs or herbal extracts in regulating ferroptosis on non-alcoholic fatty liver disease

**FIGURE 2:**
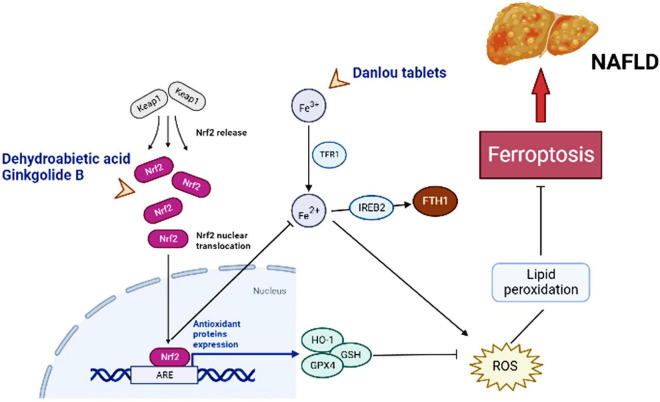
Major molecules, genes, and metabolic networks for ferroptosis involved in NAFLD interfering by traditional Chinese medicine. Keap1, kelch like ECH associated protein 1; Nrf2-ARE, nuclear factor erythroid-2-related factor-2-antioxidant reactive element; TFR1, transferrin receptor 1; IREB2, iron-responsive element binding protein 2; FTH1, ferritin heavy chain; HO-1, hame oxygenase-1; GSH, glutathione; GPx4, GSH peroxidase 4; ROS, reactive oxygen species.

**FIGURE 3:**
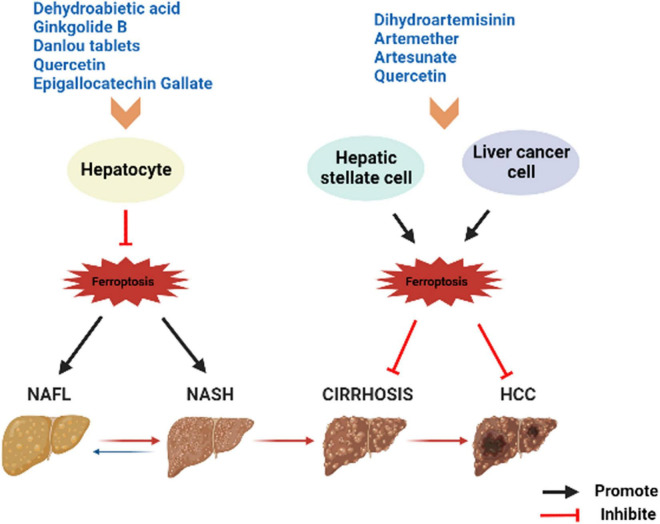
Summary of herbs or herbal extracts modulating the disease spectrum of NAFLD. NAFL, non-alcoholic fatty liver; NASH, non-alcoholic steatohepatitis; HCC, hepatocellular carcinoma.

Currently, there is no agreed standard or definitive effective drugs for the treatment of NAFLD. The commonly used chemotherapeutic drug sorafenib is resistant to treatment in patients with advanced HCC. Therefore, the search for more effective new drugs has become an urgent task. TCM occupies an equally important position as Western medicine in health management and disease treatment, and even numerous of clinical cases have proven to be superior in treatment of certain diseases (53–56). TCM is the natural treasure trove of compounds with a wide range of sources, a great deal of active ingredients, and the stability of structure (57). TCM intervention in ferroptosis has certain efficacy and value. For example, artemisinin and piperine amide are believed to exert effective mechanisms of anti-cancer by interfering ferroptosis in HCC, pancreatic cancer and other tumor diseases (58, 59). Baicalein is also a natural inhibitor of ferroptosis, weakening lipid peroxidation and ROS production and protecting cells of acute lymphoblastic leukemia induced by RSL3 from ferroptosis (60). Herbs or compounds and derived compounds or extracts have a certain ameliorating effect on NAFLD based on antioxidants, lipid metabolism and intestinal microbiota regulation (61). Therefore, TCM can intervene with NAFLD (Figure 2) or NAFLD-HCC by regulating ferroptosis (Table 1 and Figure 3).

**TABLE 1:** Intervention effects of TCM in regulating ferroptosis on NAFLD.

TCM	Function	Stage in NAFLD	References
DAA	Activates Nrf2, leading to reducing lipid peroxidation	Simple steatosis and NASH	(36, 65, 67)
GB	Activates Nrf2, leading to reducing lipid peroxidation	Simple steatosis and NASH	(70, 71)
Que	Targets Mitochondrial ROS-Mediated Ferroptosis	Simple steatosis and NASH, HCC	(75–79)
EGCG	Inhibits system Xc^–^, for preventing GSH consumption, GPx4 inactivation and lipid peroxidation	NASH	(83–85)
DLT	Increases Fe^2+^ accumulation, promoting ubiquitination of the IREB2 protein and suppressing expression of FTH1	Simple steatosis and NASH	(90)
DHA	Decreases the expression of GPx4, inducing HSC ferroptosis; Activates the anti-survival UPR and upregulates CHAC1 expression	Fibrosis, HCC	(93–95)
ART	Inhibits ubiquitination of IRP2, promoting its accumulation for HSC ferroptosis	Fibrosis	(46)
Artesunate	Activates HSC ferritinophagy/ferroptosis; Acts synergistically with sorafenib	Fibrosis, HCC	(96, 97)

ART, Artemether; CHAC1, Chac glutathione-specific γ-glutamylcyclo-transferase 1; DAA, Dehydroabietic acid; DHA, dihydroartemisinin; DLT, Danlou tablet; EGCG, Epigallocatechin Gallate; FTH1, ferritin heavy chain; GB, Ginkgolide B; HCC, hepatocellular carcinoma; HSC, hepatic stellate cell; Que, Quercetin; Nrf2, nuclear factor erythroid-2-related factor-2; ROS, reactive oxygen species; GSH, glutathione; GPx4, glutathione peroxidase; IREB2, iron-responsive element-binding protein 2; IRP2, iron regulatory protein; UPR, unfolded protein response.

### Monomers

#### Dehydroabietic acid

Dehydroabietic acid (DAA) is a natural diterpenoid resin acid, which is primarily obtained by catalytic disproportionation of rosin abietic acid (62). It is stable in nature and has the properties of anti-tumor (63) and anti-inflammatory (64). DAA alleviates insulin resistance and weakens hepatic steatosis and lipid accumulation through activation of PPAR-γ and PPAR-α in high-fat diet (HFD) model mice, leading to reducing hepatic function (65). Further studies revealed that DAA was able to bind to Keap1 in the cytoplasm and increase the luciferase activity of Nrf2-antioxidant reactive element (ARE), promoting the expression of downstream antioxidant gene hame oxygenase-1 (HO-1), GSH, and GPx4 through the Keap1/Nrf2 pathway, while DAA treatment results in achieving attenuation of ROS and lipid peroxidation (36). Importantly, Nrf2 is a necessary transcription factor that regulates the cellular oxidative stress reaction and also controls Fe^2+^, which is manifested as an inhibition of ferroptosis (27, 66). HO-1, an important source of intracellular iron, plays a key role in ferroptosis induced by erastin (67). At the same time, DAA increased the expression of key genes of ferroptosis such as ferroptosis suppressor protein 1 (FSP1) *in vivo* and *in vitro* (36). Therefore, it is believed that DAA inhibits ferroptosis through activation of the Keap1/Nrf2 pathway and is a potentially effective means of treating NAFLD. One shortcoming, however, is that these studies did not compare DAA with ferroptosis inhibitors. If available, the credibility of DAA modulation of ferroptosis to improve NAFLD would be increased.

#### Ginkgolide B

Ginkgolide B (GB) belongs to terpene trilactones and is the main active ingredient in Ginkgo biloba leaf extract (68). Like DAA, the effect of anti-inflammatory function by GB is well known (69). Application of GB for treatment of HFD-induced obese mice reduced the body weight and triglyceride (TG) levels and improved steatosis in the liver (70). To verify whether the protective effect of GB on steatosis hepatocytes is related to ferroptosis and the underlying molecular mechanisms, Yang et al. conducted *in vivo* and *in vitro* experiments (71). Mice fed with HFD and HepG2 cells treated by palmitic acid (PA) oleic acid (OA) exhibited significant reduction in Fe^2+^ concentrations and obvious increase in Fe^3+^ concentrations, and both showed changes in combinations of biomarkers based on ferroptosis, such as the upregulation of TFR1, and the inhibition of Nrf2 expression (71). The reversal effect was evident after GB administration, with a clear promotion in the expression of Nrf2, which facilitated iron metabolism and inhibited oxidative stress in the liver (71). And the effect of high concentrations of GB approximated that of the positive control drug atorvastatin, an ferroptosis inhibitor. TFR1, as an ferroptosis sensitive protein, also plays a crucial role in hepatic iron metabolism. It can be found that GB and DAA have similarities in the pathways related to the regulation of ferroptosis, both of which may mobilize downstream genes by influencing Nrf2 activity, and finally exert the role of anti-ferroptosis, while achieving a balance of iron metabolism and lipid peroxidation.

#### Quercetin

Quercetin (Que) is a polyhydroxy flavonoid widely distributed in fruits, vegetables, and the root, leave or fruit from medicinal plants. The antioxidant activity of Que is excellent, and the phenolic hydroxyl groups rich in its structure can inactivate free radicals by providing active hydrogen while being oxidized to a more stable form of free radicals themselves (72). In addition, Que is a natural iron chelator. Its B ring displays the catechol portion and multiple free hydroxyl groups that drive the reduction of Fe^3+^ to Fe^2+^ (73); and at pH 7.2, quercetin completely blocks the iron-promoted Fenton reaction at the micromolar level (74). This is an effective strategy to prevent excess iron-induced oxidative stress, which means that chelation with Fe^2+^ is the key to the antioxidant activity of Que. And it is oxidative stress that plays a crucial role in the pathogenesis of NAFLD. Zhu et al. found that Que intake resulted in a 39% reduction in hepatic TG content and a 1.5-fold increase in VLDL in HFD-induced NAFLD rats (75). Yang et al. applied Que in NAFLD model *in vivo* and *vitro* and showed a significant decrease in serum transaminase levels, a recovery for liver superoxide dismutase, catalase and GSH levels, and a relieve in lipid accumulation (76). In a randomized, double-blind and controlled trial, 90 patients supplemented with Que twice daily for 12 weeks showed significantly higher erythrocyte levels but evidently lower mean erythrocyte volume and hemoglobin, as well as ferritin compared to controls (77). It was suggested that Que exhibits potent in animal models hepatoprotective effect and also beneficial for some NAFLD-related biomarkers in clinical patients. Recently, Jiang et al. found that lipid peroxidation, lipid accumulation and ferroptosis induced by HFD could be alleviated *via* Que supplementation in mice. Next, Que was found to markedly depress mitochondrial ROS production in FFA-treated L-02 cells, with similar effects to Fer-1, an ferroptosis inhibitor (78). Mitochondrial ROS is a unique property of ferroptosis that distinguishes it from other types of PCD, and chelation of Fe^2+^ in the liver by Que reduced hepatocyte lipid peroxidation and ROS production. In addition, Que seems to have twofold properties. It was shown that for HepG_2_ hepatoma cells it was able to induce lysosomal activation mediated by transcription factor TFEB, promote ferritin degradation and eventually induce ferroptosis (79). However, the specific signaling pathway by which Que regulates mitochondrial ROS to improve NAFLD has not been elucidated, and this may be something that could be explored further.

#### Epigallocatechin gallate

Epigallocatechin Gallate (EGCG) is the main polyphenol catechin in green tea, with antioxidant and anti-inflammatory effects, and is also beneficial in metabolic syndrome and different types of liver injury (80, 81). These advantages rely on its unique structure. EGCG has three hydroxyl groups at carbons 3’, 4’, and 5’ of the B ring and an esterified gallate portion at carbon 3’ of the C ring, which contributes to its ability to scavenge free radicals and chelate transition metal ions (80). Data from multiple *in vitro* and *vivo* experiments showed that EGCG is helpful for improving NAFLD-related fibrosis and HCC (81). EGCG increases the activity of mitochondrial complex chains, thus promoting lipid peroxidation to prevent hepatic steatosis (82). EGCG-treated rat liver showed a decrease The MDA levels was significantly decrease, while GSH and SOD levels was clearly elevated in mice treated with EGCG (83), the antioxidant activity of the liver increased. In recent years, EGCG was found to be a novel ferroptosis inhibitor, which can prevent the depletion of GSH, inactivation of GPx4 and lipid peroxidation by chelating iron ion (84). Ning et al. investigated the effect of EGCG on NASH by intraperitoneal injection and gavage, and found that the iron accumulation in the liver of mice fed by both methods was significantly lower than that of the NASH group, and the long-chain fatty acid coenzyme ACSBG expression was increased, with a significant negative correlation with *Bacillus mimicus*, a representative lineage organism genus of the intestinal microbiota (85). ACSBG is necessary in fatty acid metabolism and ferroptosis pathway, indicating that EGCG altered the intestinal microbiota and regulated the metabolism of NASH mice, which in turn improved lipid accumulation and ferroptosis, so as to prevent the development of NASH. Therefore, the regulation of EGCG on NAFLD is inseparable from its efficacy as an ferroptosis inhibitor. It is also worth noting that humans may experience side effects if they ingest large amounts of EGCG, so the safe dose range needs to be considered when applying it.

### Compounds

#### Danlou tablet

Danlou tablet (DLT) are composed of ten Herbs, including Chuanqiong, Radix Salvia Miltiorrhiza, Trichosanthes, Allium macrostemon, Pueraria lobata, Paeoniae Rubra, Tulip, Rhizoma Drynariae, Alismol, and Radix Astragali (86). Among them, Alismol, Pueraria lobata, and Radix Astragali exhibit the ability to anti-oxidant stress and anti-inflammatory (87–89). Ethanol extracts of Danlou tablet (EEDT) can inhibit inflammation by downregulating the NF-κB single signal and promote the outflow of cholesterol and lipids by activating the PPARα/ABCA1 signaling pathway (90). Xin et al. established a model of NAFLD in HFD ApoE-/- mice to explore whether DLT mediates the occurrence and development of NAFLD through the ferroptosis pathway (91). After the DLT intervention, the Fe^2+^ levels in liver tissue of mice were significantly reduced, and the protein and mRNA expression levels of GPx4 and ferritin heavy chain (FTH1) were apparently elevated, and the cytoplasm was brownish yellow and dark in color (91). Moreover, the protein and mRNA expressions of iron-responsive element-binding protein 2 (IREB2) were reduced, and hepatocyte swelling was lightened, and the number of fat vacuoles was completely lessened (91). Unlike DAA and GB, IREB2 is a key indicator of cellular ferroptosis, and increased Fe^2+^ can promote ubiquitination of the IREB2 protein and suppress expression of FTH1. This is sufficient to proof that DLT targets ferroptosis to inhibit oxidative stress and inflammatory factor levels to achieve a protective effect on the liver of NAFLD model mice. The evidence on DLT still appears weak, so more verifications are necessary.

#### Artemisinin compounds

Artemisinin is the active ingredient of the dried stems and leaves of Artemisia annua, a member of the Asteraceae family, and belongs to the sesquiterpenoids. The derivatives of it include dihydroartemisinin (DHA), artemether (ART), artesunate and so on. In addition to anti-malaria, artemisinin compounds also have various pharmacological effects such as anti-inflammatory and anti-fibrotic (92). Zhang et al. found that DHA triggered ferroptosis to eliminate the activation of HSC, which characterized by iron overload, GSH depletion, lipid ROS accumulation, and peroxidation, whereas Fer-1 and Lip-1 inhibited the DHA effect (93). Shen et al. reported similar results, at the same time, they discovered that DHA attenuates liver fibrosis by activating autophagy to trigger ferroptosis in HSC (94). In addition, DHA induced ferroptosis as an antitumor agent in primary liver cancer (PLC) by activating the anti-survival unfolded protein response (UPR) and upregulating Chac GSH –specific γ-glutamylcyclo-transferase 1 (CHAC1) expression, which was significantly attenuated by Fer-1 and DFO application after iron loading (95). These reveal a potential mechanism by which DHA ameliorates liver fibrosis or PLC, and moreover suggest that ferroptosis is a favorable method to eliminate activated HSC or PLC cells. ART was detected to promote the accumulation of iron regulatory protein (IRP2) by inhibiting its ubiquitination, thus inducing an increase in iron content, generating a large amount of ROS and leading to the onset of ferroptosis of HSCs (46). Kong et al. demonstrated that artesunate obviously evoked ferroptosis of activated HSC in fibrotic liver, as characterized by decreased cell viability, accumulated iron, elevated lipid peroxidation, and diminished antioxidant capacity. In contrast, the inhibition of DFO almost abolished the antifibrotic effect induced by artesunate (96). Artesunate is a clinically well-tolerated compound that acts synergistically with sorafenib to induce ferroptosis in the HCC cell lines Huh7, SNU-449, and SNU-182 (97). Thus, artemisinin compounds have prominent effects as ferroptosis inducers to hinder HSC activation in the liver fibrosis stage of NAFLD, resulting in antifibrosis. In addition, all the above results imply a possible interaction between autophagy and ferroptosis from another perspective, which deserves to be explored in depth.

## Conclusion and perspectives

With the increasing incidence and the wide spread of complications, NAFLD has become one of the most concerned chronic liver diseases. The unclear pathogenesis is a major obstacle to the treatment of NAFLD, and at present, the main focus is to protect liver with pharmacological, especially TCM. The core steps of ferroptosis are reflected in the development of the NAFLD disease spectrum, and the manifestation at different stages may vary according to the current pathological features, which also opens a new approach for the study of the hepatic protective mechanism of TCM. Although studies on the improvement of NAFLD by intervention of ferroptosis in herbs or herbal extracts are not well reported yet, these suggest their strong potential to be used as natural ferroptosis inhibitors. Compared to classical ones, they have the advantage of being widely available, less expensive, more stable, and fewer side effects. In addition to the above mentioned, there are many studies on the protective effect of liver by herbs or herbal extracts through the means of anti-lipid peroxidation. Hesperidin has been shown in *in vitro* and *in vitro* experiments to upregulate antioxidant levels by activating the PI3K/AKT-Nrf2 pathway and alleviate liver steatosis by inhibiting NF-κB-mediated inflammation (98). Bicyclic, extracts of Wuweizi, has a wide range of pharmacological effects that attenuate tetracycline-induced hepatic steatosis, and hepatic lipid accumulation and physalide steatosis are ameliorated (99). They are all potential regulators for ferroptosis, and whether the mechanisms involved in liver protection are related to the ferroptosis pathway that ultimately led to the regulation of NAFLD should be further explored in works. For HCC, several therapies or drugs have been tried in the clinic, but no breakthroughs have been achieved, and even some approved drugs later failed to inhibit tumor growth due to the emergence of drug resistance mechanisms. Ferroptosis is considered to be the most promising tumor growth inhibitor that can affect the development and progression of HCC by regulating intracellular iron levels and ROS (100). This provides new therapeutic options for patients with HCC. The research prospect of TCM targeting ferroptosis is very promising, and although there are some undeniably limitations and difficulties involved, the meaning for the prevention and treatment of NAFLD and NAFLD-HCC is great. For example, ferroptosis is involved in the occurrence and development of NAFLD and can serve as an independent predictor of early alterations in NAFLD, as well as a potentially important target for prevention and treatment in clinical practice. However, the disadvantage is that most of the current research focuses on animal models, and there is still less evidence to prove the mechanism of ferroptosis from the molecular level or in clinical patients. Whether ferroptosis or other forms of PCD can be clearly distinguished during the disease for targeted prevention and treatment is also deserves further exploration. In addition, Studies on the regulation of ferroptosis by herbs or herbal extracts at different stages of NAFLD development to achieve intervention remains inadequate, and the related mechanisms need to be further explored as well.

## Author contributions

QW and ZC designed the study, searched the literature, and drafted the manuscript. YD and YT made the figures. YC edited the manuscript and supervised the work. All authors approved the final manuscript.

## References

[1] Dirim AB Kalayci T Guzel Dirim M Demir S Cavus B Cifcibasi Ormeci A A mysterious cause of recurrent acute liver dysfunction for over a decade. *Gastroenterol Rep.* (2022) 10:goab053. 10.1093/gastro/goab053 35382171 PMC8973007

[2] Tripodi A Lombardi R Primignani M La Mura V Peyvandi F Fracanzani AL. Hypercoagulability in patients with non-alcoholic fatty liver disease (Nafld): causes and consequences. *Biomedicines.* (2022) 10:249. 10.3390/biomedicines10020249 35203457 PMC8869363

[3] Loomba R Friedman SL Shulman GI. Mechanisms and disease consequences of nonalcoholic fatty liver disease. *Cell.* (2021) 184:2537–64. 10.1016/j.cell.2021.04.015 33989548 PMC12168897

[4] Younossi Z Tacke F Arrese M Chander Sharma B Mostafa I Bugianesi E Global perspectives on nonalcoholic fatty liver disease and nonalcoholic steatohepatitis. *Hepatology.* (2019) 69:2672–82. 10.1002/hep.30251 30179269

[5] Eslam M Newsome PN Sarin SK Anstee QM Targher G Romero-Gomez M A new definition for metabolic dysfunction-associated fatty liver disease: an international expert consensus statement. *J Hepatol.* (2020) 73:202–9. 10.1016/j.jhep.2020.03.039 32278004

[6] Tilg H Effenberger M. From Nafld to Mafld: when pathophysiology succeeds. *Nat Rev Gastroenterol Hepatol.* (2020) 17:387–8. 10.1038/s41575-020-0316-6 32461575

[7] Chrysavgis L Giannakodimos I Diamantopoulou P Cholongitas E. Non-alcoholic fatty liver disease and hepatocellular carcinoma: clinical challenges of an intriguing link. *World J Gastroenterol.* (2022) 28:310–31. 10.3748/wjg.v28.i3.310 35110952 PMC8771615

[8] Foerster F Gairing SJ Müller L Galle PR. Nafld-driven Hcc: safety and efficacy of current and emerging treatment options. *J Hepatol.* (2022) 76:446–57. 10.1016/j.jhep.2021.09.007 34555422

[9] Hester D Golabi P Paik J Younossi I Mishra A Younossi ZM. Among medicare patients with hepatocellular carcinoma, non-alcoholic fatty liver disease is the most common etiology and cause of mortality. *J Clin Gastroenterol.* (2020) 54:459–67. 10.1097/mcg.0000000000001172 30672817

[10] Chen Z Tian R She Z Cai J Li H. Role of oxidative stress in the pathogenesis of nonalcoholic fatty liver disease. *Free Radic Biol Med.* (2020) 152:116–41. 10.1016/j.freeradbiomed.2020.02.025 32156524

[11] Ke Y Xu C Lin J Li Y. Role of hepatokines in non-alcoholic fatty liver disease. *J Transl Int Med.* (2019) 7:143–8. 10.2478/jtim-2019-0029 32010600 PMC6985917

[12] Prikhodko VA Bezborodkina NN Okovityi SV. Pharmacotherapy for non-alcoholic fatty liver disease: emerging targets and drug candidates. *Biomedicines.* (2022) 10:274. 10.3390/biomedicines10020274 35203484 PMC8869100

[13] Aizawa S Brar G Tsukamoto H. Cell death and liver disease. *Gut Liver.* (2020) 14:20–9. 10.5009/gnl18486 30917630 PMC6974333

[14] Afonso MB Castro RE Rodrigues CMP. Processes exacerbating apoptosis in non-alcoholic steatohepatitis. *Clin Sci.* (2019) 133:2245–64. 10.1042/CS20190068 31742325

[15] Savic D Hodson L Neubauer S Pavlides M. The importance of the fatty acid transporter l-carnitine in non-alcoholic fatty liver disease (Nafld). *Nutrients.* (2020) 12:2178. 10.3390/nu12082178 32708036 PMC7469009

[16] Zhang H Zhang E Hu H. Role of ferroptosis in non-alcoholic fatty liver disease and its implications for therapeutic strategies. *Biomedicines.* (2021) 9:1660. 10.3390/biomedicines9111660 34829889 PMC8615581

[17] Chu Q Gu X Zheng Q Wang J Zhu H. Mitochondrial mechanisms of apoptosis and necroptosis in liver diseases. *Anal Cell Pathol.* (2021) 2021:8900122. 10.1155/2021/8900122 34804779 PMC8601834

[18] Zhao J Hu Y Peng J. Targeting programmed cell death in metabolic dysfunction-associated fatty liver disease (Mafld): a promising new therapy. *Cell Mol Biol Lett.* (2021) 26:17. 10.1186/s11658-021-00254-z 33962586 PMC8103580

[19] Tsurusaki S Tsuchiya Y Koumura T Nakasone M Sakamoto T Matsuoka M Hepatic ferroptosis plays an important role as the trigger for initiating inflammation in nonalcoholic steatohepatitis. *Cell Death Dis.* (2019) 10:449. 10.1038/s41419-019-1678-y 31209199 PMC6579767

[20] Hernandez-Alvarez MI Sebastian D Vives S Ivanova S Bartoccioni P Kakimoto P Deficient endoplasmic reticulum-mitochondrial phosphatidylserine transfer causes liver disease. *Cell.* (2019) 177:881–95 e17. 10.1016/j.cell.2019.04.010 31051106

[21] Li X Wang TX Huang X Li Y Sun T Zang S Targeting ferroptosis alleviates methionine-choline deficient (mcd)-diet induced nash by suppressing liver lipotoxicity. *Liver Int.* (2020) 40:1378–94. 10.1111/liv.14428 32145145

[22] Dai X Zhang R Wang B. Contribution of classification based on ferroptosis-related genes to the heterogeneity of Mafld. *BMC Gastroenterol.* (2022) 22:55. 10.1186/s12876-022-02137-9 35144542 PMC8830092

[23] Wu J Wang Y Jiang R Xue R Yin X Wu M Ferroptosis in liver disease: new insights into disease mechanisms. *Cell Death Discov.* (2021) 7:276. 10.1038/s41420-021-00660-4 34611144 PMC8492622

[24] Wang XH Lang R Liang Y Zeng Q Chen N Yu RH. Traditional Chinese medicine in treating iga nephropathy: from basic science to clinical research. *J Transl Int Med.* (2021) 9:161–7. 10.2478/jtim-2021-0021 34900626 PMC8629415

[25] Lang R Wang X Liang Y Yan L Shi B Yu R. Research progress in the treatment of idiopathic membranous nephropathy using traditional chinese medicine. *J Transl Int Med.* (2020) 8:3–8. 10.2478/jtim-2020-0002 32435606 PMC7227163

[26] Wu L Liu M Liang J Li N Yang D Cai J Ferroptosis as a new mechanism in parkinson’s disease therapy using traditional chinese medicine. *Front Pharmacol.* (2021) 12:659584. 10.3389/fphar.2021.659584 34163356 PMC8215498

[27] Xie Y Hou W Song X Yu Y Huang J Sun X Ferroptosis: process and function. *Cell Death Differ.* (2016) 23:369–79. 10.1038/cdd.2015.158 26794443 PMC5072448

[28] Stockwell BR Friedmann Angeli JP Bayir H Bush AI Conrad M Dixon SJ Ferroptosis: a regulated cell death nexus linking metabolism, redox biology, and disease. *Cell.* (2017) 171:273–85. 10.1016/j.cell.2017.09.021 28985560 PMC5685180

[29] Gao M Monian P Jiang X. Metabolism and iron signaling in ferroptotic cell death. *Oncotarget.* (2015) 6:35145–6. 26387139 10.18632/oncotarget.5671 PMC4742090

[30] Yang WS Stockwell BR. Ferroptosis: death by lipid peroxidation. *Trends Cell Biol.* (2016) 26:165–76. 10.1016/j.tcb.2015.10.014 26653790 PMC4764384

[31] Doll S Proneth B Tyurina YY Panzilius E Kobayashi S Ingold I Acsl4 dictates ferroptosis sensitivity by shaping cellular lipid composition. *Nat Chem Biol.* (2017) 13:91–8. 10.1038/nchembio.2239 27842070 PMC5610546

[32] Kagan VE Mao G Qu F Angeli JP Doll S Croix CS Oxidized arachidonic and adrenic pes navigate cells to ferroptosis. *Nat Chem Biol.* (2017) 13:81–90. 10.1038/nchembio.2238 27842066 PMC5506843

[33] Bridges RJ Natale NR Patel SA. System Xc(-) Cystine/glutamate antiporter: an update on molecular pharmacology and roles within the Cns. *Br J Pharmacol.* (2012) 165:20–34. 10.1111/j.1476-5381.2011.01480.x 21564084 PMC3252963

[34] Cao JY Dixon SJ. Mechanisms of ferroptosis. *Cell Mol Life Sci.* (2016) 73:2195–209. 10.1007/s00018-016-2194-1 27048822 PMC4887533

[35] Jiang L Kon N Li T Wang SJ Su T Hibshoosh H Ferroptosis as a P53-mediated activity during tumour suppression. *Nature.* (2015) 520:57–62. 10.1038/nature14344 25799988 PMC4455927

[36] Gao G Xie Z Li EW Yuan Y Fu Y Wang P Dehydroabietic acid improves nonalcoholic fatty liver disease through activating the Keap1/Nrf2-are signaling pathway to reduce ferroptosis. *J Nat Med.* (2021) 75:540–52. 10.1007/s11418-021-01491-4 33590347

[37] Kerins MJ Ooi A. The roles of Nrf2 in modulating cellular iron homeostasis. *Antioxid Redox Signal.* (2018) 29:1756–73. 10.1089/ars.2017.7176 28793787 PMC6208163

[38] Mao C Liu X Zhang Y Lei G Yan Y Lee H Dhodh-mediated ferroptosis defence is a targetable vulnerability in cancer. *Nature.* (2021) 593:586–90. 10.1038/s41586-021-03539-7 33981038 PMC8895686

[39] Friedman SL Neuschwander-Tetri BA Rinella M Sanyal AJ. Mechanisms of Nafld development and therapeutic strategies. *Nat Med.* (2018) 24:908–22. 10.1038/s41591-018-0104-9 29967350 PMC6553468

[40] Corradini E Buzzetti E Dongiovanni P Scarlini S Caleffi A Pelusi S Ceruloplasmin gene variants are associated with hyperferritinemia and increased liver iron in patients with Nafld. *J Hepatol.* (2021) 75:506–13. 10.1016/j.jhep.2021.03.014 33774058

[41] Ma W Feng Y Jia L Li S Li J Wang Z Dietary iron modulates glucose and lipid homeostasis in diabetic mice. *Biol Trace Elem Res.* (2019) 189:194–200. 10.1007/s12011-018-1446-3 30027366

[42] Backe MB Moen IW Ellervik C Hansen JB Mandrup-Poulsen T. Iron regulation of pancreatic beta-cell functions and oxidative stress. *Annu Rev Nutr.* (2016) 36:241–73. 10.1146/annurev-nutr-071715-050939 27146016

[43] Loguercio C De Girolamo V de Sio I Tuccillo C Ascione A Baldi F Non-alcoholic fatty liver disease in an area of southern italy: main clinical, histological, and pathophysiological aspects. *J Hepatol.* (2001) 35:568–74. 10.1016/s0168-8278(01)00192-1 11690701

[44] Sanyal AJ Chalasani N Kowdley KV McCullough A Diehl AM Bass NM Pioglitazone, vitamin E, or placebo for nonalcoholic steatohepatitis. *N Engl J Med.* (2010) 362:1675–85. 10.1056/NEJMoa0907929 20427778 PMC2928471

[45] Qi J Kim JW Zhou Z Lim CW Kim B. Ferroptosis affects the progression of nonalcoholic steatohepatitis via the modulation of lipid peroxidation-mediated cell death in mice. *Am J Pathol.* (2020) 190:68–81. 10.1016/j.ajpath.2019.09.011 31610178

[46] Li Y Jin C Shen M Wang Z Tan S Chen A Iron regulatory protein 2 is required for artemether –mediated anti-hepatic fibrosis through ferroptosis pathway. *Free Radic Biol Med.* (2020) 160:845–59. 10.1016/j.freeradbiomed.2020.09.008 32947011

[47] Zhang Z Yao Z Wang L Ding H Shao J Chen A Activation of ferritinophagy is required for the RNA-binding protein Elavl1/Hur to regulate ferroptosis in hepatic stellate cells. *Autophagy.* (2018) 14:2083–103. 10.1080/15548627.2018.1503146 30081711 PMC6984765

[48] Zhang Z Guo M Li Y Shen M Kong D Shao J RNA-binding protein Zfp36/Ttp protects against ferroptosis by regulating autophagy signaling pathway in hepatic stellate cells. *Autophagy.* (2020) 16:1482–505. 10.1080/15548627.2019.1687985 31679460 PMC7469536

[49] Hassannia B Vandenabeele P Vanden Berghe T. Targeting ferroptosis to iron out cancer. *Cancer Cell.* (2019) 35:830–49. 10.1016/j.ccell.2019.04.002 31105042

[50] Aydin Y Kurt R Song K Lin D Osman H Youngquist B Hepatic stress response in Hcv infection promotes Stat3-mediated inhibition of Hnf4a-Mir-122 feedback loop in liver fibrosis and cancer progression. *Cancers.* (2019) 11:1407. 10.3390/cancers11101407 31547152 PMC6827087

[51] Zhang X Wang L Li H Zhang L Zheng X Cheng W. Crosstalk between noncoding RNAS and ferroptosis: new dawn for overcoming cancer progression. *Cell Death Dis.* (2020) 11:580. 10.1038/s41419-020-02772-8 32709863 PMC7381619

[52] Wu ZY Trenner M Boon RA Spin JM Maegdefessel L. Long noncoding Rnas in key cellular processes involved in aortic aneurysms. *Atherosclerosis.* (2020) 292:112–8. 10.1016/j.atherosclerosis.2019.11.013 31785492 PMC6949864

[53] Hu Y Ye Z She Y Li L Wu M Qin K Efficacy and safety of probiotics combined with traditional chinese medicine for ulcerative colitis: a systematic review and meta-analysis. *Front Pharmacol.* (2022) 13:844961. 10.3389/fphar.2022.844961 35321324 PMC8936956

[54] Wei JJ Guo RJ Fu GJ Liang X Xu ZM Liao X Registration of intervention trials of traditional chinese medicine for four neurological diseases on chinese clinical trial registry and clinicaltrials.gov: a narrative review. *J Tradit Chin Med.* (2022) 42:148–53. 10.19852/j.cnki.jtcm.2022.01.010 35294135 PMC10164634

[55] Wang A Zhao W Yan K Huang P Zhang H Zhang Z Mechanisms and efficacy of traditional Chinese medicine in heart failure. *Front Pharmacol.* (2022) 13:810587. 10.3389/fphar.2022.810587 35281941 PMC8908244

[56] Xu J Zhang J Wang J. The application of traditional chinese medicine against the tumor immune escape. *J Transl Int Med.* (2020) 8:203–4. 10.2478/jtim-2020-0032 33511046 PMC7805287

[57] Yao CL Zhang JQ Li JY Wei WL Wu SF Guo DA. Traditional Chinese Medicine (Tcm) as a source of new anticancer drugs. *Nat Prod Rep.* (2021) 38:1618–33. 10.1039/d0np00057d 33511969

[58] Su Y Zhao D Jin C Li Z Sun S Xia S Dihydroartemisinin induces ferroptosis in Hcc by promoting the formation of Pebp1/15-Lo. *Oxid Med Cell Longev.* (2021) 2021:3456725. 10.1155/2021/3456725 34925691 PMC8683180

[59] Yamaguchi Y Kasukabe T Kumakura S. Piperlongumine rapidly induces the death of human pancreatic cancer cells mainly through the induction of ferroptosis. *Int J Oncol.* (2018) 52:1011–22. 10.3892/ijo.2018.4259 29393418

[60] Probst L Dachert J Schenk B Fulda S. Lipoxygenase inhibitors protect acute lymphoblastic leukemia cells from ferroptotic cell death. *Biochem Pharmacol.* (2017) 140:41–52. 10.1016/j.bcp.2017.06.112 28595877

[61] Dai X Feng J Chen Y Huang S Shi X Liu X Traditional Chinese medicine in nonalcoholic fatty liver disease: molecular insights and therapeutic perspectives. *Chin Med.* (2021) 16:68. 10.1186/s13020-021-00469-4 34344394 PMC8330116

[62] Kim WJ Kim W Bae JM Gim J Kim SJ. Dehydroabietic acid is a novel survivin inhibitor for gastric cancer. *Plants.* (2021) 10:1047. 10.3390/plants10061047 34067279 PMC8224772

[63] Chen NY Xie YL Lu GD Ye F Li XY Huang YW Synthesis and antitumor evaluation of (Aryl)methyl-amine derivatives of dehydroabietic acid-based B ring-fused-thiazole as potential Pi3k/Akt/Mtor signaling pathway inhibitors. *Mol Divers.* (2021) 25:967–79. 10.1007/s11030-020-10081-7 32297120

[64] Kim E Kang YG Kim YJ Lee TR Yoo BC Jo M Dehydroabietic acid suppresses inflammatory response via suppression of Src-, Syk-, and Tak1-mediated pathways. *Int J Mol Sci.* (2019) 20:1593. 10.3390/ijms20071593 30934981 PMC6480320

[65] Xie Z Gao G Wang H Li E Yuan Y Xu J Dehydroabietic acid alleviates high fat diet-induced insulin resistance and hepatic steatosis through dual activation of Ppar-Gamma and Ppar-Alpha. *Biomed Pharmacother.* (2020) 127:110155. 10.1016/j.biopha.2020.110155 32413669

[66] Dodson M Castro-Portuguez R Zhang DD. Nrf2 plays a critical role in mitigating lipid peroxidation and ferroptosis. *Redox Biol.* (2019) 23:101107. 10.1016/j.redox.2019.101107 30692038 PMC6859567

[67] Kwon M-Y Park E Lee S-J Chung SW. Heme oxygenase-1 accelerates erastin-induced ferroptotic cell death. *Oncotarget.* (2015) 6:24393–403. 10.18632/oncotarget.5162 26405158 PMC4695193

[68] Zhu PC Tong Q Zhuang Z Wang ZH Deng LH Zheng GQ Ginkgolide B for Myocardial Ischemia/Reperfusion Injury: A Preclinical Systematic Review and Meta-Analysis. *Front Physiol* (2019) 10:1292. 10.3389/fphys.2019.01292 31681006 PMC6807679

[69] Wu F Shi W Zhou G Yao H Xu C Xiao W Ginkgolide B functions as a determinant constituent of ginkgolides in alleviating lipopolysaccharide-induced lung injury. *Biomed Pharmacother.* (2016) 81:71–8. 10.1016/j.biopha.2016.03.048 27261579

[70] Luo L Li Y Wang D Zhao Y Wang Y Li F Ginkgolide B lowers body weight and ameliorates hepatic steatosis in high-fat diet-induced obese mice correlated with pregnane X receptor activation. *RSC Adv.* (2017) 7:37858–66. 10.1039/c7ra05621d

[71] Yang Y Chen J Gao Q Shan X Wang J Lv Z. Study on the attenuated effect of ginkgolide b on ferroptosis in high fat diet induced nonalcoholic fatty liver disease. *Toxicology.* (2020) 445:152599. 10.1016/j.tox.2020.152599 32976958

[72] Sharma A Kashyap D Sak K Tuli HS Sharma AK. Therapeutic charm of quercetin and its derivatives: a review of research and patents. *Pharm Pat Anal.* (2018) 7:15–32. 10.4155/ppa-2017-0030 29227203

[73] Xiao L Luo G Tang Y Yao P. Quercetin and iron metabolism: what we know and what we need to know. *Food Chem Toxicol.* (2018) 114:190–203. 10.1016/j.fct.2018.02.022 29432835

[74] Guo M Perez C Wei Y Rapoza E Su G Bou-Abdallah F Iron-binding properties of plant phenolics and cranberry’s bio-effects. *Dalton Trans.* (2007) 43:4951–61. 10.1039/b705136k 17992280 PMC2645657

[75] Zhu X Xiong T Liu P Guo X Xiao L Zhou F Quercetin ameliorates Hfd-induced Nafld by promoting hepatic Vldl assembly and lipophagy via the Ire1a/Xbp1s Pathway. *Food Chem Toxicol.* (2018) 114:52–60. 10.1016/j.fct.2018.02.019 29438776

[76] Yang H Yang T Heng C Zhou Y Jiang Z Qian X Quercetin improves nonalcoholic fatty liver by ameliorating inflammation, oxidative stress, and lipid metabolism in Db/Db Mice. *Phytother Res.* (2019) 33:3140–52. 10.1002/ptr.6486 31452288

[77] Pasdar Y Oubari F Zarif MN Abbasi M Pourmahmoudi A Hosseinikia M. Effects of quercetin supplementation on hematological parameters in non-alcoholic fatty liver disease: a randomized, double-blind, placebo-controlled pilot study. *Clin Nutr Res.* (2020) 9:11–9. 10.7762/cnr.2020.9.1.11 32095444 PMC7015726

[78] Jiang JJ Zhang GF Zheng JY Sun JH Ding SB. Targeting mitochondrial ros-mediated ferroptosis by quercetin alleviates high-fat diet-induced hepatic lipotoxicity. *Front Pharmacol.* (2022) 13:876550. 10.3389/fphar.2022.876550 35496312 PMC9039018

[79] Wang ZX Ma J Li XY Wu Y Shi H Chen Y Quercetin induces P53-independent cancer cell death through lysosome activation by the transcription factor Eb and reactive oxygen species-dependent ferroptosis. *Br J Pharmacol.* (2021) 178:1133–48. 10.1111/bph.15350 33347603

[80] Legeay S Rodier M Fillon L Faure S Clere N. Epigallocatechin gallate: a review of its beneficial properties to prevent metabolic syndrome. *Nutrients.* (2015) 7:5443–68. 10.3390/nu7075230 26198245 PMC4517007

[81] Chen C Liu Q Liu L Hu YY Feng Q. Potential biological effects of (-)-epigallocatechin-3-gallate on the treatment of nonalcoholic fatty liver disease. *Mol Nutr Food Res.* (2018) 62:1700483. 10.1002/mnfr.201700483 28799714 PMC6120134

[82] Santamarina AB Carvalho-Silva M Gomes LM Okuda MH Santana AA Streck EL Decaffeinated green tea extract rich in epigallocatechin-3-gallate prevents fatty liver disease by increased activities of mitochondrial respiratory chain complexes in diet-induced obesity mice. *J Nutr Biochem.* (2015) 26:1348–56. 10.1016/j.jnutbio.2015.07.002 26300331

[83] Tang G Xu Y Zhang C Wang N Li H Feng Y. Green tea and epigallocatechin gallate (Egcg) for the management of nonalcoholic fatty liver diseases (Nafld): insights into the role of oxidative stress and antioxidant mechanism. *Antioxidants.* (2021) 10:1076. 10.3390/antiox10071076 34356308 PMC8301033

[84] Kose T Vera-Aviles M Sharp PA Latunde-Dada GO. Curcumin and (-)- epigallocatechin-3-gallate protect murine min6 pancreatic beta-cells against iron toxicity and erastin-induced ferroptosis. *Pharmaceuticals.* (2019) 12:26. 10.3390/ph12010026 30736288 PMC6469157

[85] Ning K Lu K Chen Q Guo Z Du X Riaz F Epigallocatechin gallate protects mice against methionine-choline-deficient-diet-induced nonalcoholic steatohepatitis by improving gut microbiota to attenuate hepatic injury and regulate metabolism. *ACS Omega.* (2020) 5:20800–9. 10.1021/acsomega.0c01689 32875214 PMC7450495

[86] Wang L Wu T Si C Wang H Yue K Shang S Danlou tablet activates autophagy of vascular adventitial fibroblasts through Pi3k/Akt/Mtor to protect cells from damage caused by atherosclerosis. *Front Pharmacol.* (2021) 12:730525. 10.3389/fphar.2021.730525 34867337 PMC8637544

[87] Xu L Jing M Yang L Jin L Gong P Lu J The alisma and rhizoma decoction abates nonalcoholic steatohepatitis-associated liver injuries in mice by modulating oxidative stress and autophagy. *BMC Complement Altern Med.* (2019) 19:92. 10.1186/s12906-019-2488-6 31035991 PMC6489313

[88] Jiang ZB Gao J Chai YH Li W Luo YF Chen YZ. Astragaloside alleviates alcoholic fatty liver disease by suppressing oxidative stress. *Kaohsiung J Med Sci.* (2021) 37:718–29. 10.1002/kjm2.12390 33973356 PMC11896517

[89] Wan XM Chen J Wang M Zheng C Zhou XL. Puerarin attenuates cadmium-induced hepatic lipid metabolism disorder by inhibiting oxidative stress and inflammation in mice. *J Inorg Biochem.* (2021) 222:111521. 10.1016/j.jinorgbio.2021.111521 34171769

[90] Hao D Danbin W Maojuan G Chun S Bin L Lin Y Ethanol extracts of danlou tablet attenuate atherosclerosis via inhibiting inflammation and promoting lipid effluent. *Pharmacol Res.* (2019) 146:104306. 10.1016/j.phrs.2019.104306 31181336

[91] Xin Z Wen-na C Nan S Jing-xuan Z Yu-tong L. Danlou tablets attenuate oxidative damage of liver in mice with non-alco?holic fatty liver disease through ferroptosis pathway. *Chin J Pathophysiol.* (2021) 37:2180–8.

[92] Jiang YQ Dong YJ Zhou FJ Chen JP Zhou YT Tian CW Research progress on artemisinin and its derivatives. *Chin Tradit Herbal Drugs.* (2022) 53:599–608.

[93] Zhang Z Wang X Wang Z Zhang Z Cao Y Wei Z Dihydroartemisinin alleviates hepatic fibrosis through inducing ferroptosis in hepatic stellate cells. *Biofactors.* (2021) 47:801–18. 10.1002/biof.1764 34129254

[94] Shen M Guo M Li Y Wang Y Qiu Y Shao J M(6)a methylation is required for dihydroartemisinin to alleviate liver fibrosis by inducing ferroptosis in hepatic stellate cells. *Free Radic Biol Med.* (2022) 182:246–59. 10.1016/j.freeradbiomed.2022.02.028 35248719

[95] Wang Z Li M Liu Y Qiao Z Bai T Yang L Dihydroartemisinin triggers ferroptosis in primary liver cancer cells by promoting and unfolded protein response-induced upregulation of chac1 expression. *Oncol Rep.* (2021) 46:240. 10.3892/or.2021.8191 34558645 PMC8485000

[96] Kong Z Liu R Cheng Y. Artesunate alleviates liver fibrosis by regulating ferroptosis signaling pathway. *Biomed Pharmacother.* (2019) 109:2043–53. 10.1016/j.biopha.2018.11.030 30551460

[97] Li ZJ Dai HQ Huang XW Feng J Deng JH Wang ZX Artesunate synergizes with sorafenib to induce ferroptosis in hepatocellular carcinoma. *Acta Pharmacol Sin.* (2021) 42:301–10. 10.1038/s41401-020-0478-3 32699265 PMC8026986

[98] Li J Wang T Liu P Yang F Wang X Zheng W Hesperetin ameliorates hepatic oxidative stress and inflammation Via the Pi3k/Akt-Nrf2-are pathway in oleic acid-induced Hepg2 cells and a rat model of high-fat diet-induced Nafld. *Food Funct.* (2021) 12:3898–918. 10.1039/d0fo02736g 33977953

[99] Yao XM Li Y Li HW Cheng XY Lin AB Qu JG. Bicyclol attenuates tetracycline-induced fatty liver associated with inhibition of hepatic Er stress and apoptosis in mice. *Can J Physiol Pharmacol.* (2016) 94:1–8. 10.1139/cjpp-2015-0074 26640164

[100] Couri T Pillai A. Goals and targets for personalized therapy for Hcc. *Hepatol Int.* (2019) 13:125–37. 10.1007/s12072-018-9919-1 30600478

